# Berberine potently attenuates intestinal polyps growth in ApcMin mice and familial adenomatous polyposis patients through inhibition of Wnt signalling

**DOI:** 10.1111/jcmm.12119

**Published:** 2013-09-09

**Authors:** Junfang Zhang, Hailong Cao, Bing Zhang, Hanwei Cao, Xiuqin Xu, Hang Ruan, Tingting Yi, Li Tan, Rui Qu, Gang Song, Bangmao Wang, Tianhui Hu

**Affiliations:** a Cancer Research Center, Xiamen University Medical CollegeXiamen, China; b School of life Science, Xiamen UniversityXiamen, China; c Department of Gastroenterology General Hospital, Tianjin Medical UniversityTianjin, China; d Department of Basic Medicine, Xiamen University Medical CollegeXiamen, China; e Institute of Stem Cell and Regenerative Medicine, Xiamen University Medical CollegeXiamen, China

**Keywords:** berberine, colon cancer, Wnt, signal transduction, familial adenomatous polyposis

## Abstract

As a traditional anti-inflammatory Chinese herbal medicine, Alkaloid berberine has been recently reported to exhibit anti-tumour effects against a wide spectrum of cancer. However, the mechanism was largely unknown. Gene chip array reveals that with berberine treatment, c-Myc, the target gene of Wnt pathway, was down-regulated 5.3-folds, indicating that berberine might inhibit Wnt signalling. TOPflash analysis revealed that Wnt activity was significantly reduced after berberine treatment, and the mechanism of which might be that berberine disrupted β-catenin transfer to nucleus through up-regulating the expression of adenomatous polyposis coli (APC) gene and stabilized APC-β-catenin complex. Berberine administration in ApcMin/+ mice exhibited fewer and smaller polyps in intestine, along with reduction in cyclin D1 and c-Myc expression. In clinical practice, oral administration of berberine also significantly reduced the familial adenomatous polyposis patients' polyp size along with the inhibition of cyclin D1 expression in polyp samples. These observations indicate that berberine inhibits colon tumour formation through inhibition of Wnt/β-catenin signalling and berberine might be a promising drug for the prevention of colon cancer.

## Introduction

Colorectal cancer is the second largest cause of cancer-related deaths in Western countries 1. Colorectal cancer arises from the colorectal epithelium as a result of the accumulation of genetic alterations in defined oncogenes and tumour suppressor genes 2. Co-ordinated Wnt signalling is necessary to maintain the delicate balance between proliferation and differentiation 3. The adenomatous polyposis coli (APC) gene encodes the adenomatous polyposis coli tumour suppressor protein, germline mutation of which characterizes familial adenomatous polyposis (FAP) 4, an autosomal intestinal cancer syndrome. The syndrome is characterized by hundreds of adenomatous colorectal polyps, with an almost inevitable progression to colorectal cancer at an average age of 35–40 5. Most patients are asymptomatic for years until the adenomas are large and numerous, and cause rectal bleeding or even anaemia, or cancer develops. Generally, cancers start to develop a decade after the appearance of the polyps. Inactivation of APC is also recognized as the key early event in the development of sporadic colorectal cancers, and its loss results in constitutive activity of the β-catenin, and to the constitutive activation of Wnt signalling 6. Wnt induces stabilization of cytosolic β-catenin, which is associated with T-cell factors (TCFs) in the nucleus, leading to the expression of specific target genes, including c-Myc and cyclin D1 7. β-catenin, a key effector of Wnt signalling, promotes cell proliferation by inducing gene transcription through the activation of TCF/LEF transcription factors 6. c-Myc is the critical mediator of the early stages of neoplasia following Apc loss 4. The levels of cytoplasmic β-catenin are normally controlled by a multi-proteins destruction complex that targets β-catenin for degradation in the proteasome 1. The canonical mechanism of the regulation of β-catenin involves its phosphorylation by casein kinase 1 at the Ser-45 site and by glycogen synthase kinase 3β (GSK3β) at the Thr-41, Ser-37 and Ser-33 sites. This phosphorylation targets β-catenin to ubiquitination and degradation by the proteasomal degradation system. In addition to phosphorylation by Casein Kinase I (CK1) and GSK3β at the N terminus, β-catenin can be phosphorylated by protein kinase A (PKA) at two novel sites, Ser-552 and Ser-675, and thus promotes the transcriptional activity of TCF/LEF 8.

Berberine was initially isolated from the herbs Rhizoma coptidis (Chinese name Huang-Lian), which belongs to the camptothecin family of drugs, and has been used as a traditional Chinese medicine to treat various infectious disorders for more than 3000 years 9. It has been reported that berberine can be used as an anti-diarrhoea, anti-hypertension, anti-arrhythmia and anti-inflammatory agent 10–13. Additionally, this natural product was demonstrated to possess anti-tumour activity. Animal studies have shown that berberine can suppress chemical-induced carcinogenesis, clastogenesis, tumour promotion and tumour invasion, and also it is a radiosensitizer of tumour cells, but not of normal cells 14–17. However, how berberine mediates these effects is not fully understood. Many studies have reported the *in vitro* and *in vivo* anti-cancer effects of berberine through different mechanisms and it was reported that the anti-tumour effects may vary, depending on the types of cancer cells, the duration of treatment and the doses 17–21. Previous reports have shown the tumour suppression activities of berberine through different mechanisms, including modulating the activities of many proteins including myeloid cell leukemia-1, cyclooxygenase-2 22, multi-drug resistance-1, tumour necrosis factor α (TNF-α), inducible nitric oxide synthase 14, interleukin-12 and AM-1 23–27.

The tumour-suppressing effect of berberine has been implicated to be related to its anti-inflammation functions. Recently, more and more reports have shown that chronic inflammation might induce tumourigenesis 28–29. Berberine can effectively inhibit both chronic colon inflammation and colon tumour formation. But how these two processes are related is largely unknown. It is well known that Wnt signalling is necessary to maintain the delicate balance between proliferation and differentiation, and is closely related to inflammation and colorectal tumourigenesis. The possible relationship between berberine and Wnt signal pathway remains a very intriguing topic. The aim of the present study was to investigate the effect of berberine on the Wnt signalling and illustrate the molecular mechanisms governing this effect.

## Materials and methods

### Cell lines and reagents

Three human colon cancer cell lines, KM12C, KM12SM and KM12L4A, kindly provided by Professor I. J. Fidler (M.D. Anderson Cancer Center, Houston, TX, USA), were used as models in the present study. The parental cell line KM12C was originally established from a colon carcinoma Dukes' B2. Cells from this cell line were repeatedly injected into the caecum and spleen in athymic mice, to form two new cell lines KM12L4a and KM12L4a, both with high metastatic potential. The cell lines were maintained in Eagle's MEM medium supplemented with 10% heat-inactivated foetal bovine serum (FBS), sodium pyruvate, vitamins and a cocktail of penicillin and streptomycin at 37°C in 5% carbon dioxide (Invitrogen/Gibco, Paisley, UK). Berberine was purchased from Sigma-Aldrich (St. Louis, MO, USA) and was dissolved in dimethyl sulfoxide (DMSO) to a final concentration of 0.1% DMSO in the medium, and the medium with 0.1% DMSO was used as the control. Antibodies against β-actin, β-catenin, APC and horseradish peroxidase-linked anti-rabbit or mouse IgG were purchased from Santa Cruz Biotechnology (Santa Cruz, CA, USA). Antibodies against cyclin D1, cyclin B1, CDC-2, c-Myc, tubulin, lamin B and Phospho-β-catenin were purchased from Cell Signaling Technology (Beverly, MA, USA).

### Cell proliferation assay

The effect of berberine on cell proliferation was tested using CHEMICON's BrdU cell proliferation assay kit according to the manufacturer's protocol 30.

### Western Blot

Samples were collected by lysing cells in RIPA lysis buffer [50 mM Tris, pH 7.4, 150 mM NaCl, 1 mM ethylenediaminetetraacetic acid (EDTA), 0.1% SDS, 1% TritonX-100, 1% sodium deoxycholate, and 1 mM phenylmethylsulfonyl fluoride (PMSF)]. Each sample (80 mg of cellular proteins) was size-fractionated using SDS-PAGE and electrotransferred onto polyvinylidene difluoride (PVDF) transfer membranes (Dupont, Boston, MA, USA). Blots were incubated for 1 hr at room temperature in 5% bovine serum albumin for blocking, and proteins were detected with primary antibodies overnight and then blotted with horseradish peroxidase conjugated secondary antibodies for 1 hr. The immunoblots were visualized with ECL (GE Healthcare, Bucks, UK).

### TCF/LEF-luciferase reporter assay

The TOPflash firefly luciferase and TK-RL Renilla luciferase constructs were used to measure the activation of the Wnt pathway after berberine treatment. Cells grown on 24-well plates were transfected in quadruplicates with cDNAs (20 ng/well) for TCF-luciferase reporter (TOPflash) along with a control Renilla plasmid (TK-RL) by Lipofectamine 2000 transfection Reagent (Invitrogen, Carlsbad, CA, USA). Twenty-four hours post-transfection, the cells were treated with different concentrations of berberine. Six hours later, the cells were lysed and the luciferase activity was measured and normalized to the corresponding Renilla activity with the dual-luciferase assay kit (Promega, Madison, WI, USA).

### Co-immunoprecipitation (co-IP)

For IP studies, cellular extracts were normalized for protein concentration and pre-cleared for 1 hr at 4°C with 0.4 ml of protein A-coated Sepharose beads. Immunoprecipitation was carried out at 4°C by incubating the fractions for 2 hrs with an anti-APC antibody or anti β-catenin antibody, and then for 1 hr with 0.4 ml of protein A/G-Sepharose beads. Control experiments were performed in the presence of the immunizing peptides, or with control IgG antisera. The IPs proteins were recovered by boiling the Sepharose beads in SDS sample buffer.

### Quantitative real-time PCR

The mRNA levels of APC were evaluated by quantitative real-time PCR. Total RNA was extracted with TRIzol Reagent (Invitrogen) and treated with DNase I at 37°C for 30 min. The cDNA was synthesized from 1 mg of total RNA using 15 U MMLV reverse transcriptase (Invitrogen) in 20 ml of a reaction mixture containing 0.5 mg oligo 31, 18 primers, 40 U recombinant RNase inhibitor (Takara, Dalian, China), reverse transcription buffer and 1 mM of each dNTP (Takara). The reaction was incubated at 37°C for 50 min., followed by 70°C for 5 min. and 4°C for 5 min. The cDNA samples were diluted 10-fold with nuclease-free water. Real-time PCR was then performed in an Opticon machine (MJ Research, Waltham, MA, USA) using SYBR Green I Dye (Takara), with glyceraldehyde-3-phosphate dehydrogenase (GAPDH) as reference, and the results were evaluated with Opticon Monitor software. Primer sequences for APC were upstream 5′-GAC ATT TTG TTT CAA ATG AAA CTT T-3′ and downstream 5′-TTG GGA TGG GAT GCT ACT TT. For GAPDH, the primers were upstream 5′-TCC TGC ACC ACC AAC TGC TTA G-3′ and downstream 5′-AGT GGC AGT GAT GGC ATG GAC T-3′.

### Immunohistochemistry

Tumour tissue specimens were fixed in neutral formalin and embedded in paraffin after collection from the killed mice. Tissue sections 5 μm thick were dewaxed and incubated with 0.01 M natrium citricum for antigen retrieval. The slides were rinsed in phosphate-buffered saline and incubated overnight at 4°C with diluted anti-cyclinD1 or anti-c-Myc antibodies. Following steps were then performed with the immunostaining kit according to the manufacturer's instructions.

### *In vivo* experiment

Animal experiments were conducted in accordance with the regulations of experimental animal administration and the animal ethical committee of the Medical College of Xiamen University. C57BL/6J-ApcMin/+ and wild-type C57BL/6J animals were purchased from Model Animal Research Center of Nanjing University. For polyp/tumour scoring and the establishment of survival curves, all animals received high nutrient food (Teklad #2019 extruded, Harlan Winkelmann, Borchen, Germany) and water, whereas mating pairs were fed on standard mouse chow. All the mice were housed in a temperature-controlled environment (20–22°C) with a 12:12 hr light:dark cycle.

### Oral administration of berberine

Seven FAP patients who were diagnosed at the Digestive Endoscopy Center of Tianjin General Hospital, Tianjin Medical University between January 2008 and December 2012 were investigated. The mean age was 28.71 ± 6.82 years old (range: 15–36). Five were males, and two were females. Because of worries about the cardiovascular side effects and the cost of long-term use, they refused to take celecoxib, which is commonly used for FAP in Western countries. Moreover, all patients did not want to have their entire colorectum removed. All patients had a clean colon and rectum at study entry. At the baseline endoscopy, the number and size of the polyps were evaluated, and then all of the polyps below 10 cm from the anal margin were removed. After 6 months, a colonoscopy was taken and the polyps recurred to almost the same level as 6 months before. The colonoscopy result at this stage was used as control group (without berberine treatment).Then the polyps were removed again and then the patients took berberine treatment (300 mg, three times per day) immediately following polypectomy. The patients were followed up and asked to undergo a colonoscopy after 6 months and the result was used as berberine administration group. Evaluations at base line and month six included a medicine taking, physical examinations, colonoscopy appearances and immunohistochemistry of β-catenin and cyclinD1 with biopsies of the polyps before and after berberine administration. Informed consents for colonoscopy and the present study were granted from all the patients before the procedure and ethical committee approval was obtained from Tianjin General Hospital Ethics Committee.

### Microarray analysis

Illumina HumanRef-8 Expression BeadChips were used for array hybridization (Illumina, San Diego, CA, USA) according to manufacturer's protocols. Briefly, total RNA (2 μg) from untreated cells and from berberine-treated cells at each time point was converted to biotinylated cDNA with the Illumina RNA Amplification Kit (Ambion, Austin, TX, USA). Samples were purified using the RNeasy kit (Qiagen, Valencia, CA, USA). Hybridization to the Sentrix HumanRef-8 Expression BeadChip, washing and scanning were performed according to the Illumina Bead Station 500× manual. Data were extracted, normalized and background subtracted using Illumina BeadStudio software. Transcript signals above 0.95 were accepted as presence of expression.

### Statistical analysis

All values are expressed as the means ± SD. Statistical analysis was performed by Dunnett's test. Differences were considered significant when the *P* < 0.01.

## Results

### Berberine could transfer into the nucleus *via* passive diffusion, and potently attenuates colorectal cancer growth

**Figure 1:**
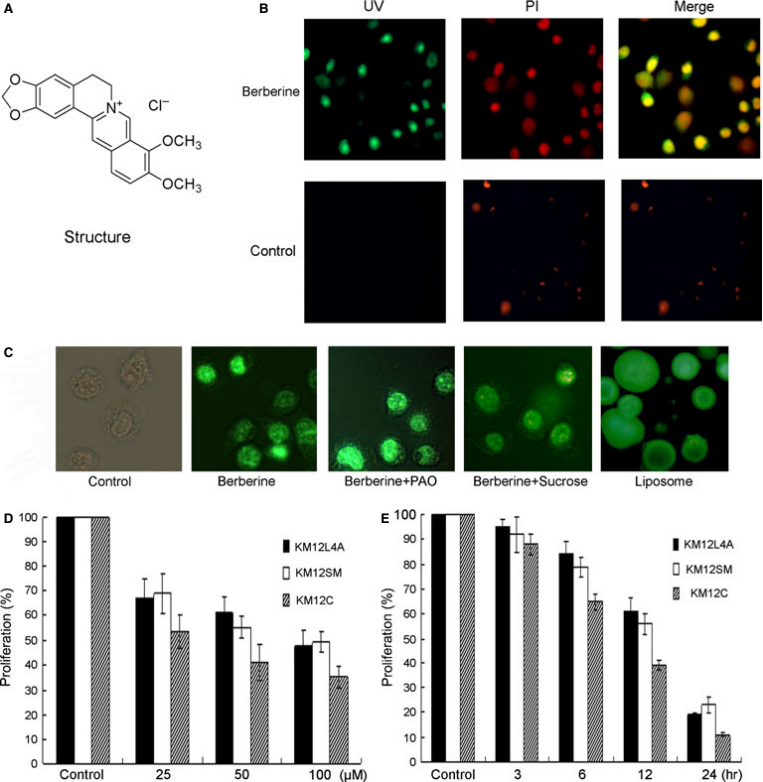
Cellular location of berberine and its anti-proliferation effect. (A) The structure of berberine. (B) KM12C were treated with 100 μM berberine for 12 hrs, and the cells exhibited green colour under UV. The green fluorescence was concentrated at the centre of cells and overlapped with the red nuclear staining of Prodidium Iodide, indicating that berberine is located in the nucleus. (C) Neither PAO nor hypertonic sucrose changed the berberine membrane transport. When berberine and liposome were mixed together, liposome exhibited green fluorescence under UV. (D) The effect of berberine on cell proliferation was tested by BrdU cell proliferation assay kit. *P < 0.01, **P < 0.05.

Figure 1A is the structure of berberine. It is important to determine berberine's cellular location before study of its anti-cancer mechanisms. Berberine has absorption in the UV-A region at 342 nm and emits at 550 nm, suggesting that it is visible under fluorescent microscope. Figure 1B demonstrates that after berberine treatment, the KM12C exhibited green fluorescence in the nuclear area. This signal was overlapped with the red Propidium iodide staining, demonstrating that berberine can be concentrated in the nucleus. Phenylarsine Oxide (PAO) and hypertonic sucrose were reported to inhibit carrier-mediated drug transport process 32–33; however, neither PAO nor hypertonic sucrose inhibited berberine transporting into the cell (Fig. 1C). Liposome, comparable to the cellular membranes composed of amphiphilic phospholipids, was mixed with berberine, and it was found that liposome glowed green fluorescence, illustrating that berberine could enter the liposome (Fig. 1C). Therefore, berberine is most likely transported across biological membranes *via* passive diffusion.

Uncontrolled cell proliferation is a hallmark of cancer. We asked whether berberine had any effect on the cell proliferation. Using the CHEMICON's BrdU Cell Proliferation Assay Kit, we quantitatively studied newly synthesized DNA of actively proliferating cells. Berberine treatment reduced the ratio of newly synthesized DNA in dosage- and time-dependent manners (Fig. 1D).

### Gene chip array of berberine treated cells

Several studies from our and other laboratories have indicated that berberine can interact with DNA and regulate gene expression 34. To further study how genes were regulated by berberine, we did a gene chip analysis on berberine-treated KM12C cells and analysed those mRNAs with more than 1.5-fold change after the berberine treatments for 2, 6 and 12 hrs, and found that 469 mRNAs were down-regulated, and 373 mRNAs were up-regulated. Among those genes, there were many DNA-binding proteins whose expressions were altered by berberine treatment, suggesting the underlying mechanism of berberine in modifying gene expression. Then, we analysed about 50 cancer-related genes, and found that some crucial genes regulating cell growth, cell cycle and cell apoptosis were altered by berberine, which is coincident with many previous reports on berberine's anti-cancer effects. Most of the cancer-related gene expression levels were altered about twofolds. Among those genes, the target gene of Wnt pathway, c-Myc changed most dramatically (down-regulated 5.3-fold). Other Wnt pathway-related genes such as CD44, PPARG, PCDHA12, NIN, FZD2 were also regulated. The gene chip results suggest that berberine may regulate WNT pathway. As aberrant activation of Wnt signalling represents the key initiating event for intestinal tumourigenesis, berberine might inhibit tumour growth by regulation of Wnt pathway. For this reason, we commenced to detect the effects of berberine on this pathway.

### Berberine reduces the WNT activity by blocking β-catenin's translocation to the nucleus

**Figure 2:**
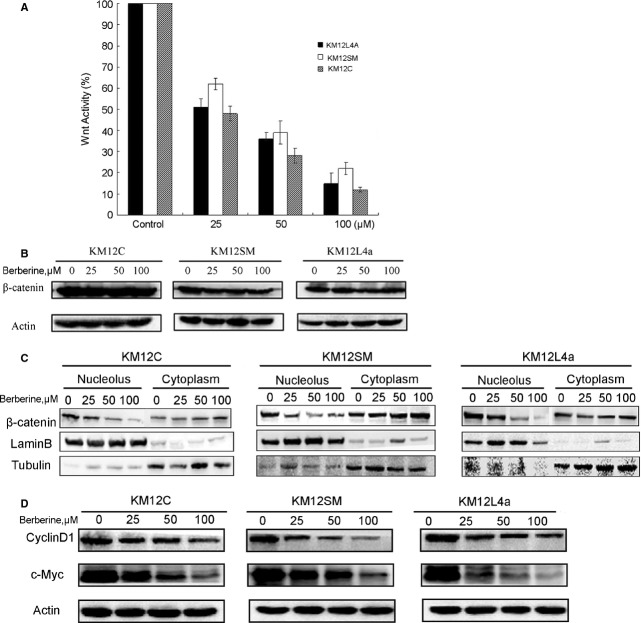
Berberine reduces the Wnt activity by blocking β-catenin's translocation. (A) Three colorectal cancer cell lines were grown on 24-well plates and transfected in quadruplicates with cDNAs (100 ng/well) for TOPflash along with a control tk-RL by Lipofectamine 2000 Transfection Reagent. 24 hrs after transfection, cells were treated with 0, 25, 50 or 100 μM berberine for 4 hrs, and then the luciferase activity was measured as described under ‘Materials and Methods’. (B) Cells were treated with berberine for 24 hrs and the levels of total β-catenin were tested by western blot. (C) Cells were treated with berberine for 24 hrs and the levels of β-catenin in the nucleus and cytoplasm were tested by western blot. Lamin B and α-tubulin were used as the internal control of nucleus and cytoplasm respectively. (D) Cells were treated with berberine for 24 hrs and the downstream target genes of Wnt signalling, cyclin D1 and c-Myc were tested.

To investigate the effect of berberine on Wnt/β-catenin signal pathway, TCF/LEF luciferase reporter assay was carried on. Twenty-four hours post-transfection, cells were treated with 0, 25, 50 and 100 μM beberine for 4 hrs, and then the luciferase activity was measured. The Wnt activity was suppressed significantly after treatment with berberine, compared with control in KM12C cells (Fig. 2A). As β-catenin plays a central role in the Wnt signalling, the level of β-catenin was tested by western blot. Beberine did not change the amount of total β-catenin after being treated for 24 hrs (Fig. 2B). However, the nuclear β-catenin was significantly decreased in a dose-dependent way, whereas the level of β-catenin in the cytoplasm was increased (Fig. 2C), indicating that berberine can block β-catenin's nuclear translocation. Figure 2D demonstrates that both cyclin D1 and c-Myc, the downstream target genes of Wnt, were down-regulated.

### Berberine up-regulated APC

**Figure 3:**
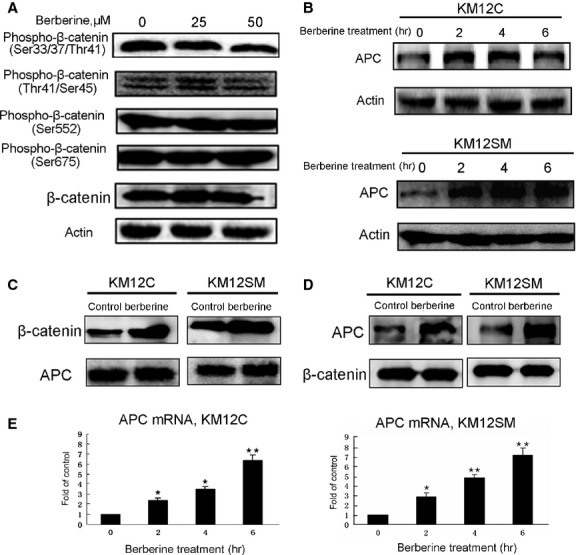
Berberine does not increase the level of phospho-β-catenin, but up-regulates APC and stabilizes the APC-β-catenin complex. (A) KM12SM cells were treated with 25 or 50 μM berberine for 12 hrs, and then the phosphorylations of different sites on β-catenin were measured. (B) The protein levels of APC were measured by western blotting after 25 μM berberine treatment for 2–6 hrs. (C) Co-IP studies were performed to determine whether berberine treatment could increase the interaction between APC and β-catenin. Cells were treated with 25 μM berberine for 6 hrs and were lysed. APC was immuno-precipitated by anti-APC antibody and western blotted with anti-β-catenin antibody (upper panel) or anti-APC antibody (lower panel). (D) Reciprocally, β-catenin was immuno-precipitated by β-catenin antibody and western blotted with anti-APC antibody (upper panel) and anti-β-catenin antibody (lower panel). (E) The level of APC mRNA after 25 μM berberine treatment for 2–6 hrs was measured by quantitative real-time PCR.

The levels of cytoplasmic β-catenin are normally controlled by a multi-proteins destruction complex to promote phosphorylation of β-catenin, which is required to trigger ubiquitination of β-catenin and its subsequent degradation by proteasomes. As β-catenin metabolism is tightly regulated through its phosphorylation, we investigated some major phosphorylation sites of β-catenin. Figure 3A shows that the phospho-β-catenin level of different sites did not increase after treatment of 25 or 50 μM berberine for 12 hrs, indicating that berberine might not affect the phosphorylation of β-catenin. As APC is an important tumour suppressor binding β-catenin, we then tested if berberine might affect the level of APC. Figure 3B shows that the protein level of APC was up-regulated after 25 μM berberine treatment for 2–6 hrs. To further evaluate the effects of berberine on the interaction between APC and β-catenin, co-immunoprecipitation studies were performed to determine whether berberine treatment could increase the interaction between APC and β-catenin. β-catenin was immunoprecipitated by APC after 25 μM berberine treatment for 6 hrs (Fig. 3C), and APC was immunoprecipitated by β-catenin after 25 μM berberine treatment for 6 hrs (Fig. 3D). Blots were detected with anti- β-catenin and anti-APC antibody. Figure 3C and D shows that berberine treatment could increase β-catenin binding to APC. Figure 3E further indicates that berberine could up-regulate the mRNA level of APC. These results suggest that berberine might prevent β-catenin from nuclear translocation by up-regulation of APC.

### Berberine reduces polyp formation and Wnt activity in Apc^Min/+^ mice

**Figure 4:**
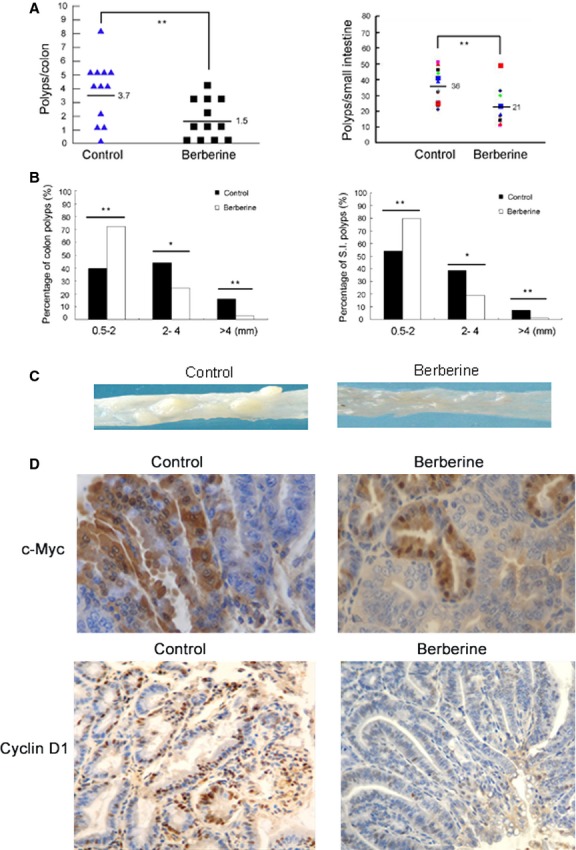
Berberine reduces polyps formation and Wnt activity in Apc^Min/+^ mice. All ApcMin/+mice received a standardized elevated fat/protein diet after weaning. ApcMin/+ mice (*n* = 12) started receiving berberine (dissolved in drinking water, 0.33 g/l) from 4 weeks old, and were killed at the 24th week. (A) Tumour numbers detected in the colon and small intestine of Apc^Min/+^ mice. (B) Size distribution of the colon and small intestine polyps. (C) Macrophotograph of the representative colons of berberine administration group and control group. (D) The expression of cyclin D1 and c-Myc in the intestinal tissues. *P < 0.01, **P < 0.05.

To further clarify the anti-cancer effect and molecular mechanism, we studied tumour development and Wnt activity in Apc^Min/+^ (multiple intestinal neoplasia) mouse model with berberine treatment. Because food composition can strongly influence intestinal tumour formation in men and mice 35, for this study, all animals received a standardized elevated fat/protein diet after weaning. Four-week-old Apc^*Min/+*^ mice (*n* = 12) began to receive berberine (dissolved in drinking water, 0.33 g/l), and killed at 24 weeks. Compared with the control group, berberine administration significantly reduced the number of polyps in colon and small intestine (Fig. 4A). Only 38% of colon polyps in berberine administration group were bigger than 2 mm in diameter, whereas in the control group, 60% of polyps were big adenomas (>2 mm in diameter). Consistent result was found in small intestinal polyps (Fig. 4B and C). Tumours were then evaluated for histopathological features. Quantification of Figure 4D demonstrated that the rates of c-Myc and cyclin D1-positive cells were reduced to 62% and 46% respectively (*P < 0.05) by berberine treatment, compared with control groups.

### Berberine reduces polyps formation and recurrence in familial adenomatous polyposis patients

**Figure 5:**
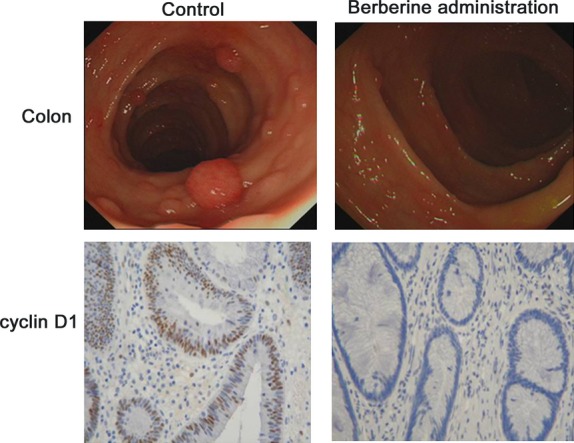
Berberine reduces polyps formation in familial adenomatous polyposis patients. Clinical colonoscopic graphs were taken from patients with berberine treatment. The upper panel shows the polyps in the colon from the same patient before and after berberine administration (as described in materials and methods). The lower panel demonstrates the expression of cyclin D1 in the corresponding polyps tissues.

Seven patients took berberine treatment immediately following polypectomy. The colonoscopy results between control and berberine administration (as described in materials and methods) are shown in Figure 5 upper panel. There were no colorectal polyp recurrences below 10 cm from the anal margin. The polyps number and size were also significantly decreased in the other part of the colon after berberine administration. Quantification of immunohistochemical staining showed that berberine decreased cyclinD1-positive cells by 68% compared in polyps between before and after berberine treatment (*P < 0.05, Fig. 5 lower panel).

## Discussion

Controlling gene expression with small DNA-binding molecules has been a challenge at the interface of medicinal chemistry and biology. Our study demonstrated that berberine could enter the nucleus, suggesting its gene regulation function. The studies that followed showed that berberine could inhibit colorectal cancer cells proliferation by inhibition of Wnt activity. We then studied how berberine interfered with WNT/β-catenin signalling. The result shows that berberine could disrupt the nuclear translocation of β-catenin. β-catenin is a key effector of Wnt signalling, promoting cell proliferation by inducing gene transcription through the activation of TCF/LEF transcription factors 6. The proto-oncogene c-Myc has been identified as a target of the Wnt pathway, and promotes cell growth and transformation by regulating the expression of the genes involving cell proliferation 36. It was reported that c-Myc deletion rescues APC deficiency in the small intestine, by which c-Myc is recognized as the critical mediator of the early stages of neoplasia following APC loss 4. Our results indicated that berberine significantly reduced the expression of c-Myc both *in vitro* and *in vivo*. Further study demonstrated that berberine increased the expression of APC. APC has been renowned as a negative regulator of WNT/β-catenin signalling. APC loss is an early event in tumourigenesis, and causes an increase in nuclear β-catenin and its transcriptional activity. This is thought to be the driving force for colon tumourigenesis. As a tumour suppressor, APC controls β-catenin function in transcription 37 and thus blocks the nuclear translocation of β-catenin. There was another report which showed that berberine might inhibit Wnt signalling by inhibition of β-catenin expression 38, which was slightly different from our conclusions as we did not observe any β-catenin down-regulation by berberine. The reason might be that berberine may have long-term effect on down-regulation of gene transcription 39, so when cells were treated with berberine for 24–72 hrs, many genes were down-regulated, including β-catenin. Previous report shows that berberine can bind to the promoter region of genes and up-regulate protein expression in short-term 9. We showed berberine could enter the nucleus, and it was highly possible that berberine might bind to the promoter region to activate the transcription of APC gene in the cells.

Many studies have reported that berberine exhibits its tumour-suppressing effect through inducing apoptosis 40, and we show here that berberine regulates the Wnt pathway. Apoptosis and cell proliferation are linked by cell-cycle regulators and are tightly controlled. Controlled and co-ordinated Wnt signalling is necessary to maintain the delicate balance between proliferation and differentiation. Aberrancies in Wnt signalling have been implicated in multiple disease processes, most notably in cancer. In colon cancer cells, Wnt activity is aberrant, thus breaks the cell homoeostasis. Association between Wnt signalling pathway and apoptosis has become increasingly established through recent reports. Genes in both apoptotic and Wnt pathways are activated in a successive co-ordinated fashion throughout embryonic development. Inappropriate activation of Wnt signalling promotes cell survival and inhibits cell death. Wnt signalling is associated with COX-2 and Prostaglandin E production, which, in turn, inhibits apoptosis in colon cancer 41. Blockade of Wnt-1 signalling induces apoptosis in human colorectal cancer cells 42. Wnt/β-catenin signalling suppressed apoptosis by inhibiting c-Myc-induced release of cytochrome c and caspase activation, and both cyclooxygenase 2 and WISP-1 were identified as effectors of the Wnt-mediated anti-apoptotic signal 43. Inhibition of Wnt signalling induces apoptosis *in vitro* and *in vivo* 44–45.

Our preliminary study showed that berberine maybe play a role in chemoprevention of intestinal adenomas in FAP patients. It may also inhibit the Wnt signalling pathway, which was in accordance with the animal and *in vitro* experiment. Our current study was non-randomized controlled trial with a rather small sample size (seven patients). This limitation was possibly because of the rare diseases of FAP. Thus, randomized controlled trial from multiple centres with a large sample size is needed to confirm the chemoprevention efficacy of berberine.

In conclusion, our study indicates that berberine might be a promising drug in prevention and treatment of colon cancer through inhibition of Wnt signalling.
